# IL-22 mRNA Expression in Blood Samples as a Useful Biomarker for Assessing the Adverse Health Effects of PCBs on Allergic Children

**DOI:** 10.3390/ijerph9124321

**Published:** 2012-11-27

**Authors:** Mayumi Tsuji, Toshihiro Kawamoto, Chihaya Koriyama, Fumio Matsumura

**Affiliations:** 1 Department of Environmental Toxicology, University of California Davis, One Shields Ave, Davis, CA 95616, USA; Email: tsuji@med.uoeh-u.ac.jp; 2 Department of Environmental Health, University of Occupational and Environmental Health, 1-1 Iseigaoka, Yahatanishi-ku, Kitakyusyu 807-8555, Japan; Email: kawamott@med.uoeh-u.ac.jp; 3 Department of Epidemiology and Preventive Medicine, Kagoshima University Graduate School of Medical and Dental Sciences, 8-35-1 Sakuragaoka, Kagoshima 890-8520, Japan; Email: fiy@m.kufm.kagoshima-u.ac.jp

**Keywords:** childhood allergy, biomarkers, environmental pollutants, IL-22, PCB

## Abstract

To facilitate the assessment of adverse effects of very low concentrations of air pollutants on general populations, we planned to establish a reliable biomarker that is also useful in identifying vulnerable populations. For this purpose we monitored several inflammation markers in blood samples from 2 year old Japanese children (N = 30), and found that those children living close to major highways (<50 m) show higher levels of mRNA expression IL-22 in their blood samples than those living further away (+50 m). This tendency was more pronounced among subjects showing positive IgE against egg and milk. We further examined association between IL-22 mRNA expression and PCB residues and found a number of significant positive correlations between each individual PCB congener and IL-22 expression. To identify the most vulnerable population among those children we selected asthma as a typical allergy-related disease, and could show that there are significant differences in the levels of IL-22 mRNA expression between IgE negative non-asthmatic subject and asthmatic children showing positive IgE reaction toward egg or milk, again. These observations support our main conclusion that IL-22 expression is a sensitive biomarker which is useful in identifying sub-populations of children who are especially vulnerable to air pollution.

## 1. Introduction

While it is widely accepted that persistent environmental pollutants such as PCBs pose a health hazard to people, particularly on vulnerable populations such as young children [1,2] and the elderly [3], it has been very difficult to obtain any epidemiological proof of direct associations between exposure to low levels of air pollution and definite adverse health effect signs. The main epidemiologic methodologies in studying the health effects of environmental pollutants have involved finding correlations between pollutant residues present in air, water, foods or in certain compartments of the body and the incidences of the specific allergic disease studies [4,5]. While the above type of approaches have been very helpful in providing many informative pieces of evidence indicating the deleterious effects of some of serious cases of environmental pollution, such as those involving accidental and occupational contamination by lead, arsenic and mercury (*i.e.*, point source) [6,7], it has been difficult to apply the same approach to widely distributed (*i.e.*, non-point source) low concentrations of persistent organic pollutants such as PCBs and dioxins which are frequently found among ordinary citizens, although some associations between exposure to these pollutants and the risk of human diseases have been suggested previously [8,9]. This is because the health effects of pollution are actually the end-result of complex interactions between toxic chemicals and physiological conditions of vulnerable tissue components of the body, which are further influenced by both the genetic composition of those individuals and their environmental variables, such as their diets and home environments [10,11]. In approaching this study, we hypothesized that hyper-allergic predisposition, especially those as showing specific IgE positive reactions, may be one of the key physiological conditions that could make those children more susceptible to the adverse health effects of PCBs.

To test the above hypothesis we have set as our primary objective to identify and establish health relevant biomarkers in whole blood samples from children of Kumamoto (Japan) that are indicative of definite adverse health effects of environmental pollutants on children, particularly those who are especially vulnerable to air pollution.

## 2. Materials and Methods

### 2.1. Subjects

Subjects were recruited between January 2009 and February 2010. We performed a questionnaire survey of 203 preschool children whose age was under 4 years old after their guardians agreed to participate in our study. Non-fasting venous blood was collected from all the children who were not infected with any diseases during the month preceding the interview. Inclusion of children into the asthma group was decided when the study subjects satisfactorily met at least two out of three criteria as follows: (1) have been told by their primary physicians that they have asthma; (2) have experienced shortness of breath and/or wheezing; (3) have received treatments for asthma or asthmatic bronchitis. Finally, case subjects (N = 15) were selected according to the above criteria. Control subjects (N = 15) were selected, on the other hand, without matching on any other factors to the subjects selected for asthma group. They were selected from children without any history of symptoms or signs related to asthma or atopy. The mean of age (SE) of all of our study subjects (N = 30) was 22.7 (1.8) months old. Mean ages (SE) for the control and asthmatic subjects were 20.5 (2.8) months and 24.9 (2.3) months, respectively [12]. The data of PCB assessments and CAP radio-allergosorbent test (CAP-RAST) to examine responses to egg, milk, and wheat are same as our previous study [12]. Review boards of Kumamoto University and the University of Occupational and the Environmental Health have approved the study proposal, as well as the manner in which informed consent was obtained from the subjects’ guardians.

### 2.2. PCBs Assessment

The amount of serum which we were able to collect for the PCB measurements was about 1 mL for each child. After blood samples were collected, the blood was allowed to clot for approximately 3 hours and then spun at 10,000 rpm for 30 minutes to separate the serum portion of the blood. The serum was transferred to clean tubes and stored at −80 °C until they were shipped to the Environmental Idea Research Laboratory (Kankyo-Sozo), Inc, which analyzed individual PCB congener levels that were quantifiable by a HRGC-HRMS method. Each PCB’s limit of detection (LOD, in pg/g-wet) was as follows: #74 + #61 = 0.09; #99 = 0.1; #118 = 0.2; #138 = 0.3; #146 = 0.4; #153 = 0.4; #156 = 0.2; #163 + #164 = 0.3; #170 = 0.6; #177 = 0.4; #178 = 0.5; #180 + #193 = 0.4; #183 = 0.5; #187 + #182 = 0.5; #194 = 0.4; #199 + #198 = 0.5. If a PCB isomer was not detected, we added 0 when we calculated the total PCB concentration [12].

### 2.3. Quantitative Real-Time PCR (q-RT-PCR)

The basic methodology of qRT-PCR has been published previously [12,13]. Primers not previously described were: for IL-10: 5′-TGGGGGAGAACCTGAAGAC-3′ (forward), 5′-CCTTGCTCTTGTTTTCACAGG-3′ (reverse); for IL-17A: 5′-ATATTGGGGCTTGCCTTTCT-3′ (forward), 5′-GTGTAATTCCAGGGGGAGGT-3′ (reverse); for IL-22: 5′-ACAGCAAATCCAGTTCTCCAA-3′ (forward), 5′-TCCAGAGGAATGTGCAAAAG-3′ (reverse); for Foxp3: 5′-CAAGGGCCAAGGAAGGG-3′ (forward), 5′-CCAGGCTGATCCTTTTCTGT-3′ (reverse); for SOCS3: 5′-ATCCTGGTGACATGCTCCTC-3′ (forward), 5′-CAAATGTTGCTTCCCCCTTA-3′ (reverse); for RelB: 5′-TCCCAACCAGGATGTCTAGC-3′ (forward), 5′-AGCCATGTCCCTTTTCCTCT-3′ (reverse).

### 2.4. Statistical Methods

The expression levels of mRNA between groups of food-specific IgE-negative and -positive were compared by Mann-Whitney U test. All analyses were performed by STATA Version 10 (Stata Corporation, College Station, TX, USA), and all *p* values presented are two-sided (α = 0.05).

## 3. Results

In an initial effort to identify biomarkers which would correctly indicate the influence of environmental factors on all study subjects, we assessed association between living near major roads and the expression of 10 different mRNA markers. Among them two markers, IL-22 and CYP1A1, showed significant correlations to the closeness of the location of their homes to major highways (*i.e.*, <50 m or +50 m; Table 1).

When all subjects were divided into two sub-groups based on food-specific IgE negative or positive (egg and milk and wheat) as judged by the expression of specific IgE in their serum, significant IL-22 correlations were recognized among the IgE-positive children, but not in the IgE-negative sub-group, suggesting the possibility of the latter being the susceptible sub-population, responding to the environmental risk factors associated with major highways. To identify the specific type of hyper-allergic predispositions that significantly influences the expression of each biomarker, we divided all 30 study subjects into two sub-groups according to whether they showed positive or negative IgE reactions to either egg, milk or wheat, and assessed the association between IgE reaction and expression of the 10 different mRNA markers to gain a perspective on the relative importance of IL-22 as a biomarker (Table 2). The results showed that IL-22 turned out to be the only marker which consistently showed a significant influence of positive IgE reaction of children to either egg- or milk-specific IgE reaction, but not to wheat.

Since air pollutants include PCBs [14,15] and subjects living close to major highways also showed high serum residues of PCBs (pg/g-wet) [+50 m; median = 480, 95% confidence interval (CI): 291, 742, <50 m; median = 548, 95%CI: 312, 1018, *p* = 0.512], in analyzing associations between PCBs and IL-22 expression, we first adopted the toxicologically accepted method of analyzing the dose-dependent effects of this class of pollutants [16] by dividing the all subjects into three sub-groups (e.g., tertiles) according to their serum levels of total PCBs. When the association between IL-22 expression and the serum level of total PCB congeners were assessed among all children showing egg-specific IgE (Table 3A) or milk-specific IgE (Table 3B), the only significant difference was found in milk-specific IgE group with the high levels of serum PCB residues (*p* = 0.028).

To increase the chance of finding significant correlations between IL-22 and PCBs, we then adopted the method of assessing their correlations to individual PCB congeners [12], instead of total PCBs. In addition, since in the above cases significant differences were most consistently detected among subjects classified into the high tertile group, we repeated the same type of analysis on subjects showing milk-specific IgE (Table 4). In this manner, a higher frequency of correlations between IL-22 and individual congeners was found among those showing positive allergic IgE reactions to milk.

When the same approach was applied to sub-group of children showing egg-specific IgE (Table 5), it was found that, significant associations between IL-22 expression and exposure to PCB congeners are detected in subjects showing “low” and “high” tertiles, but not in “medium” tertile.

**Table 1:** Relationship between the expression of select biomarkers and the distance from the home to large roads among all study subjects as well as among two sub-groups separated according to food-specific IgE status.

	All			Negative (egg and milk and wheat)			Positive (egg and/or milk and/or wheat)		
Primer	+50 m (N = 22)	<50 m (N = 8)	*p* value	+50 m (N = 6)	<50 m (N = 2)	*p* value	+50 m (N = 16)	<50 m (N = 6)	*p* value
	median (95%CI)	median (95%CI)		median (95%CI)	median (95%CI)		median (95%CI)	median (95%CI)	
IL6	1.25 (0.99, 2.69)	1.85 (1.14, 5.24)	0.348	0.89 (0.45, 12.9)	1.57 (0.90, 2.25)	0.739	1.28 (1.07, 2.73)	1.85 (1.27, 6.46)	0.238
IL8	1.02 (0.75, 1.81)	1.65 (0.81, 3.95)	0.174	1.45 (1.00, 2.34)	1.17 (0.63, 1.71)	0.505	0.96 (0.59, 2.06)	2.03 (0.96, 6.46)	0.090
IL10	2.52 (1.63, 3.33)	2.00 (0.95, 2.54)	0.205	2.88 (1.05, 7.77)	1.74 (1.32, 2.17)	0.317	2.43 (1.57, 3.53)	2.00 (0.35, 2.68)	0.461
IL17	1.47 (1.23, 1.56)	1.69 (1.09, 2.34)	0.425	0.73 (0.30, 1.71)	1.19 (1.18, 1.20)	0.505	1.51 (1.28, 2.13)	1.86 (0.98, 2.46)	0.302
IL22	28.3 (9.51, 56.9)	119 (42.8, 318)	**0.010**	2.39 (0.95, 33.1)	54.6 (53.0, 56.2)	0.046	47.8 (21.1, 90.8)	231 (27.9, 325)	**0.023**
COX2	0.59 (0.42, 1.01)	0.79 (0.29, 1.00)	0.888	0.97 (0.21, 1.16)	0.50 (0.11, 0.89)	0.182	0.49 (0.39, 0.95)	0.79 (0.41, 1.11)	0.269
CYP1A1	6.86 (1.75, 8.28)	14.2 (7.33, 32.5)	**0.010**	1.25 (0.65, 6.83)	11.6 (9.92, 13.2)	0.046	7.32 (3.57, 12.4)	16.0 (5.74, 40.4)	0.065
Foxp3	1.55 (1.24, 1.86)	1.72 (1.07, 2.03)	0.673	0.72 (0.38, 1.88)	1.12 (1.10, 1.14)	0.505	1.61 (1.29, 2.24)	1.82 (1.07, 2.22)	0.507
SOCS3	1.57 (1.27, 1.78)	1.81 (1.11, 2.08)	0.425	0.71 (0.36, 1.85)	1.18 (1.15, 1.20)	0.505	1.65 (1.31, 2.21)	1.91 (1.10, 2.30)	0.338
RelB	1.61 (1.29, 1.86)	1.85 (1.10, 2.13)	0.512	0.71 (0.36, 1.95)	1.14 (1.14, 1.15)	0.505	1.67 (1.34, 2.28)	1.92 (1.11, 2.35)	0.376

*p* values were obtained by Mann-Whitney U test; N = 30.

**Table 2:** Study on the influence of specific allergic status of all thirty children on the expression of several biomarkers as assessed by comparing children showing positive IgE reaction versus negative reactions, specifically to egg, milk or wheat.

	Egg					Milk			Wheat		
primer	all (N = 30)	negative (N = 12)	positive (N = 18)	*p* value		negative (N = 22)	positive (N = 8)	*p* value	negative (N = 22)	positive (N = 8)	*p* value
	median (95%CI)	median (95%CI)	median (95%CI)			median (95%CI)	median (95%CI)		median (95%CI)	median (95%CI)	
IL6	1.34 (1.14, 2.26)	1.19 (0.79, 6.29)	1.41 (1.21, 2.56)	0.582	1.43 (1.13, 2.80)		1.32 (0.84, 3.69)	0.639	1.43 (1.19, 2.69)	1.19 (0.63, 6.82)	0.482
IL8	1.33 (0.95, 1.79)	1.21 (0.64, 1.79)	1.40 (0.91, 2.36)	0.612		1.16 (0.75, 1.71)	2.03 (0.77, 4.58)	0.189	1.16 (0.69, 1.71)	2.37 (0.85, 4.78)	0.083
IL10	2.26 (1.79, 2.58)	2.35 (1.35, 3.81)	2.26 (1.68, 2.57)	0.612		2.17 (1.53, 3.33)	2.41 (1.41, 2.62)	0.963	2.35 (1.53, 3.33)	2.14 (1.26, 2.53)	0.453
IL17	1.50 (1.25, 1.67)	1.22 (0.51, 1.99)	1.55 (1.31, 1.69)	0.352		1.54 (1.20, 1.70)	1.42 (0.75, 2.17)	0.888	1.55 (1.25, 1.77)	1.28 (0.35, 1.79)	0.159
IL22	47.6 (22.0, 76.6)	12.3 (1.56, 55.8)	59.1 (27.4, 130)	**0.022**		28.3 (9.51, 56.9)	91.3 (35.5, 314)	**0.031**	33.2 (9.51, 62.9)	78.6 (23.4, 314)	0.111
COX2	0.68 (0.42, 0.94)	0.81 (0.32, 1.09)	0.59 (0.41, 0.91)	0.612		0.59 (0.41, 0.95)	0.74 (0.41, 1.75)	0.399	0.63 (0.42, 0.95)	0.68 (0.37, 1.75)	0.673
CYP1A1	7.50 (4.03, 11.8)	5.03 (1.24, 12.9)	8.17 (5.47, 14.4)	0.271		7.21 (2.20, 12.8)	7.92 (3.89, 25.0)	0.639	7.74 (3.23, 13.3)	6.54 (0.72, 22.2)	0.639
Foxp3	1.61 (1.26, 1.85)	1.20 (0.51, 1.88)	1.68 (1.33, 1.88)	0.352		1.66 (1.14, 1.87)	1.50 (0.82, 2.03)	0.851	1.74 (1.25, 1.89)	1.29 (0.37, 1.76)	0.146
SOCS3	1.65 (1.29, 1.81)	1.25 (0.49, 1.85)	1.71 (1.33, 1.93)	0.352		1.71 (1.20, 1.85)	1.52 (0.83, 2.08)	0.815	1.76 (1.30, 1.87)	1.30 (0.34, 1.83)	0.146
RelB	1.67 (1.29, 1.87)	1.24 (0.49, 1.95)	1.73 (1.37, 1.92)	0.374		1.72 (1.15, 1.91)	1.57 (0.84, 2.13)	0.888	1.81 (1.33, 1.93)	1.31 (0.36, 1.87)	0.146

*P* values were obtained by Mann-Whitney U test; N = 30.

**Table 3A:** Association between egg specific IgE and IL-22 as analyzed by total PCBs tertile groups.

total PCBs tertile	negative (N)	median (95%CI)	positive (N)	median (95%CI)	*p* value
low	6	6.33 (1.20, 132)	4	81.4 (30.4, 140)	0.088
medium	2	54.6 (53.0, 56.2)	8	41.3 (15.2, 278)	1.000
high	4	18.7 (1.00, 326)	6	57.6 (26.0, 293)	0.286

**Table 3B:** Association between milk specific IgE and IL-22 as analyzed by total PCBs tertile groups.

total PCBs tertile	negative (N)	median (95%CI)	positive (N)	median (95%CI)	*p* value
low	9	17.5 (3.61, 136)	1	78.8 (78.8, 78.8)	0.602
medium	8	54.6 (15.2, 278)	2	62.7 (21.6, 104)	1.000
high	5	24.2 (1.00, 61.7)	5	154 (42.1, 326)	0.028

*P* values were obtained by Mann-Whitney U test; N = 30.

**Table 4:** Association between milk specific IgE and IL-22 by individual PCBs high concentration group.

PCB	negative (N)	median (95%CI)	positive (N)	median (95%CI)	*p* value
#61 + 74	5	26.1 (1.00, 61.7)	5	154 (42.1, 326)	0.076
#99	5	24.2 (1.00, 313)	5	53.5 (21.6, 326)	0.347
#118	6	25.2 (1.03, 61.1)	4	104 (21.6, 326)	0.201
#138	4	18.7 (1.00, 61.7)	6	104 (21.6, 324)	0.088
#146	5	24.2 (1.00, 61.7)	5	154 (42.1, 326)	0.028
#153	5	24.2 (1.00, 61.7)	5	177 (42.1, 327)	0.028
#156	5	9.70 (1.00, 61.7)	5	104 (42.1, 308)	0.028
#163 + 164	4	18.7 (1.00, 61.7)	6	129 (43.3, 324)	0.033
#170	4	18.7 (1.00, 61.7)	6	129 (43.3, 324)	0.033
#177	4	18.7 (1.00, 61.7)	6	129 (43.3, 324)	0.033
#178	4	18.7 (1.00, 61.7)	6	129 (43.3, 324)	0.033
#180 + 193	4	18.7 (1.00, 61.7)	6	129 (43.3, 324)	0.033
#183	5	24.2 (1.00, 61.7)	5	154 (42.1, 326)	0.028
#182 + 187	5	24.2 (1.00, 61.7)	5	104 (42.1, 308)	0.028
#194	5	36.0 (1.00, 61.7)	5	104 (42.1, 308)	0.076
#198 + 199	5	24.2 (1.00, 61.7)	5	104 (42.1, 308)	0.028

*P* values were obtained by Mann-Whitney U test; N = 10.

**Table 5:** Association between egg specific IgE and IL-22 by individual PCBs concentration groups.

PCB	negative (N)	median (95%CI)	positive (N)	median (95%CI)	*p* value
individual **PCB low** concentration group					
#156 *	6	6.33 (1.20, 132)	5	83.9 (30.4, 313)	0.045
#182 + 187	6	6.33 (1.20, 132)	4	81.4 (30.4, 140)	0.088
#194	5	7.02 (0.95, 145)	5	140 (30.4, 313)	0.047
#198 + 199	5	7.02 (0.95, 145)	5	140 (30.4, 313)	0.047
individual **PCB medium** concentration group					
#156 **	3	56.2 (53.0, 326)	6	25.2 (18.2, 240)	0.197
#182 + 187	3	56.2 (53.0, 326)	7	26.2 (12.2, 297)	0.305
#194	4	54.6 (3.44, 326)	6	22.9 (10.5, 78.2)	0.394
#198 + 199	4	54.6 (3.44, 326)	6	23.9 (10.5, 81.2)	0.670
individual **PCB high** concentration group					
#156	3	1.33 (1.00, 36.0)	7	61.7 (19.9, 260)	0.030
#182 + 187	3	1.33 (1.00, 36.0)	7	61.7 (19.9, 260)	0.030
#182 + 187	3	1.33 (1.00, 36.1)	7	61.7 (29.8, 260)	0.030
#194	3	1.33 (1.00, 36.2)	7	61.7 (45.7, 260)	0.017
#198 + 199	3	1.33 (1.00, 36.3)	7	61.7 (29.8, 260)	0.030

*P* values were obtained by Mann-Whitney U test; N = 10, * N = 11, ** N = 9.

**Figure 1:**
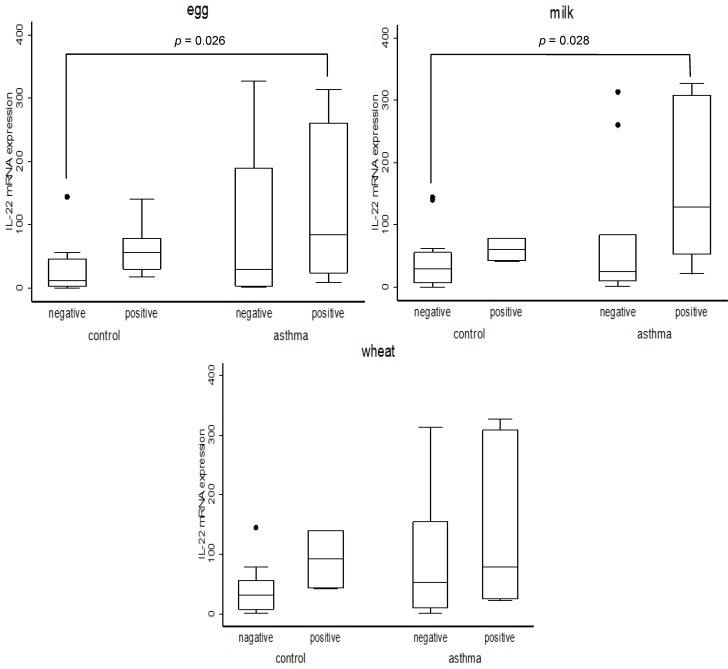
Association between IL22 and allergen specific IgE. Numbers of each category subject are as follows.Egg: control and egg specific IgE negative (N=8), control and egg specific IgE positive (N=7), asthma and egg specific IgE negative (N=4), asthma and egg specific IgE positive (N=11). Milk: control and milk specific IgE negative (N=13), control and milk specific IgE positive (N=2), asthma and milk specific IgE negative (N=9), asthma and milk specific IgE positive (N=6). Wheat: control and wheat specific IgE negative (N=13), control and wheat specific IgE positive (N=2), asthma and wheat specific IgE negative (N=9), asthma and wheat specific IgE positive (N=6). *p* values were obtained by Mann-Whitney U test.

To address the question, apart from the influence of PCBs, what else would make certain subjects ultra-sensitive to pollutants to overexpress IL-22, we assessed the possibility of asthmatic children being hyper-responsive to many environmental stress. For this purpose we have divided all subjects into four subgroups according to their asthmatic condition and to their IgE status (Figure 1).

The results indeed showed that the expression of IL-22 is significantly higher in the subgroup of asthmatic children showing the positive IgE reaction to either milk, or to egg, when the asthmatic subjects showing IgE positive reaction were directly compared to non-asthmatic ones showing negative IgE. At the same time it was noticed that in all cases the level of IL-22, which is intimately associated with lung inflammation, was always higher within each IgE positive sub-group than that in the corresponding IgE negative one, although the differences were not always statistically significant. Such an IL-22 enhancing effect of IgE was clearly exacerbated among asthmatic children.

## 4. Discussion

The major objective of this study has been to identify an appropriate biomarker indicating the association between children’s exposure to PCBs, man-made pollutants, and their expression of food specific IgEs. To this end we could identify IL-22 expression in their blood samples to be the best candidate among the 10 markers tested. Knowing that IL-22 has frequently been associated with allergic diseases [17,18,19,20,21,22], it is not totally surprising that we found its association with egg- and milk-specific IgE in this study. However, none of the above studies addressed the possibility of an IL-22 association with PCBs. A natural question arising from the above consideration is whether such an association between PCB and IL-22 expression means a direct action of PCB to cause allergic reactions, or else represents its action in PCB-susceptible populations who are pre-disposed to exhibit inflammatory responses. In this regard, it is important to consider the reports by Van Den Heuvel *et al.* [23] and Noakes *et al*. [24] who found that PCBs, at background levels, do not appear to directly affect the etiology of allergic diseases such as rhinitis, among children [25]. Furthermore, in our previous study [12], we could not find any significant association between the incidence of childhood asthma and that of rhinitis or atopic dermatitis, two typical allergic diseases, asmong the same study subjects as the current study (data not shown). Together the above information argues against the possibility of PCBs acting as direct inducers of allergic diseases. As for the second possibility of PCBs worsening the symptoms of allergy-related diseases, the two hit model of pathogenesis of asthma based on the experimental evidence from non-human primate studies [26] provides the simplest explanation. According to this scenario, the initial allergic sensitization such as that induced by children’s exposure to house dust mites, makes them predisposed to develop lung re-structuring by the deleterious actions of many air pollutants (such as ozone, pesticides and environmental tobacco smokes), leading to the eventual pathogenesis of childhood asthma. Such basic information supports the second possibility that children who are already showing allergic sensitization to food allergens likely represent the vulnerable sub-population readily reacting to air pollutants.

As for the justification of our approach of choosing individual PCB congeners, rather than the total PCBs, in obtaining positive correlations, the most important consideration is that total PCBs is not a consistently reliable parameter, since the serum PCB congener levels and compositions are greatly affected by the source of intake, the subsequent routes of PCB metabolism, transport (including transport to embryos through placenta) as well as residue re-distributions among body compartments. During the post-exposure processing the proportion of the metabolically labile congeners continue to decrease, leaving those most persistent congeners behind, which represent more reliable parameters. Aside from the above theoretical reasons, there are indeed good precedents: e.g., although Zheng *et al.* [27,28] could not find any correlations between breast cancer and exposure to the total sum or anti-estrogenic congeners of PCB, Demers *et al* [29] could detect a positive association, when they adopted specific congener analysis, which showed that three congeners (#99, 118 and 156) were specifically associated. The rationale of analyzing correlations to each congener is to help us to tease out the most significant association through well accepted epidemiologic methods of analysis. Whether the congener that consistently shows significant associations would be the one causing food specific IgE positive reactions, or not, however, must be tested in an animal model under controlled environment by testing each purified congener.

Another approach that helped us in finding statistically significant correlations was the used of a “tertile” approach, which led us to focus on the high PCB exposure group to find the best correlations. It must be pointed out that air pollution includes PCBs, polycyclic aromatic hydrocarbons (PAHs), nitro-PAHs, 1-Nitropyrene (1-NP) and so on [14,15]. In other words, PCBs may not be the only factor affecting the increased IL-22 expression in subgroup of subjects living near major highways. However, the important point is that significant associations between IL-22 mRNA expression and PCBs are found only among children showing positive IgE reactions to milk and egg (*i.e.*, a food allergy).

As for the relationship between IL-22, food-specific IgE and asthma the data in Figure 1 indicated that with respect to the expression of IL-22 in blood samples, significant differences were consistently found between those asthmatic children showing positive IgE reaction toward egg and milk, and the sub-group of non-asthmatic children showing no specific IgE reaction but not those showing positive IgE toward wheat. Although this set of data does not address the influence of PCBs, we have previously shown that, among the same study subjects as the current study, that the serum levels of PCBs in the whole asthmatic subjects are consistently higher than those in non-asthmatic ones [12]. While much more work would be needed to firmly establish the link between PCBs and IL-22 among asthmatic children, the current study shows that IL-22 is a useful marker in identifying the children showing positive IgE reaction toward milk and egg as an ultra-sensitive group.

In conclusion we could show that, among a number of potential biomarkers tested, IL-22 expression is particularly useful in detecting the effect of air pollution components, especially PCB congeners, on a sub-population of vulnerable children who exhibit positive IgE responses to milk as well as to egg. It is well known that IL-22 is produced by Th17 cells and is required to develop allergic airway inflammation, and that IL-22 production has been reported to plays a supportive role in the pathogenesis of asthma [30].
